# High expression of Ras-related protein 1A promotes an aggressive phenotype in colorectal cancer via PTEN/FOXO3/CCND1 pathway

**DOI:** 10.1186/s13046-018-0827-y

**Published:** 2018-07-31

**Authors:** Liguo Liu, Xuebing Yan, Dapeng Wu, Yi Yang, Mengcheng Li, Yang Su, Wenchao Yang, Zezhi Shan, Yuping Gao, Zhiming Jin

**Affiliations:** 1 Department of General Surgery, Shanghai Jiao Tong University Affiliated Sixth People’s Hospital, No. 600, Yi-shan Road, Shanghai, 200233 China; 2 Department of Oncology, Shanghai Jiao Tong University Affiliated Sixth People’s Hospital, No. 600, Yi-shan Road, Shanghai, 200233 China; 3 Department of Oncological Surgery, Kunshan Traditional Chinese Medicine, Hospital Affiliated to Nanjing University of Chinese Medicine, Kunshan, 215300 Jiangsu China; 4 Department of Assisted Reproduction, Xinhua Hospital, School of Medicine, Shanghai Jiaotong University, Shanghai, 200092 People’s Republic of China

**Keywords:** Colorectal cancer, RAP1A, FOXO3, Prognosis, Biomarker

## Abstract

**Background:**

Colorectal cancer (CRC) is a commonly diagnosed digestive malignancy worldwide. Ras-related protein 1A (RAP1A) is a member of the Ras superfamily of small GTPases and has been recently identified as a novel oncoprotein in several human malignancies. However, its specific role in CRC remains unclear.

**Method:**

In this study, we firstly analyzed its expression and clinical significance in a retrospective cohort of 144 CRC patients. Then, cellular assays in vitro and in vivo were performed to clarify its biological role in CRC cells. Finally, microarray analysis was utilized to investigate the molecular mechanisms regulated by RAP1A in CRC progression.

**Results:**

Firstly, RAP1A expression was abnormally higher in CRC tissues as compared with adjacent normal tissues, and significantly correlated tumor invasion. High RAP1A expression was an independent unfavourable prognostic factor for CRC patients. Combining RAP1A expression and preoperative CEA level contributed to a more accurate prognostic stratification in CRC patients. Secondly, knockdown of RAP1A dramatically inhibited the growth of CRC cells, while it was opposite for RAP1A overexpression. Finally, the microarray analysis revealed RAP1A promoted CRC growth partly through phosphatase and tensin homolog (PTEN)/forkhead box O3(FOXO3)/cyclin D1(CCND1) signaling pathway. FOXO3 overexpression could partly mimic the inhibitory effect of RAP1A knockdown in CRC growth. Moreover, FOXO3 overexpression inhibited CCND1 expression, but had no impact on RAP1A and PTEN expression.

**Conclusion:**

RAP1A promotes CRC development partly through PTEN/FOXO3 /CCND1 signaling pathway. It has a great potential to be an effective clinical biomarker and therapeutic target for CRC patients.

**Electronic supplementary material:**

The online version of this article (10.1186/s13046-018-0827-y) contains supplementary material, which is available to authorized users.

## Background

Colorectal cancer (CRC) is one of the most common digestive malignancies with approximately 1.4 million new cases and 693,900 CRC-specific deaths in 2012 worldwide [1, 2]. In the United States, it is ranked as the third most common cancer in both genders and estimated to account for 50,260 cancer deaths in 2017, according to the latest report from the American Cancer Society [3]. Although inspiring technical advances has been achieved in risk screening, early diagnosis and individualized treatment during the past decade, a considerable proportion of CRC patients are initially diagnosed with distant metastasis, with a poor 5-year survival rate ranging from 13.3–14% [3]. Furthermore, patients within localized stage are also likely to undergo tumor recurrence postoperatively and application of adjuvant chemotherapy is still controversial among these patients [4, 5]. This dilemma may be largely attributed to the fact that CRC is a complicated multi-step process involving various molecular events but few molecular biomarkers are currently available for clinical management [6]. Therefore, it is an urgent task to get a comprehensive knowledge about the underlying molecular features of CRC and identify more potential biomarkers used for disease diagnosis and treatment.

The superfamily of small GTPases plays a crucial role in signal transduction and participates in diverse biological processes such as cell proliferation, division and differentiation [7]. It contains five functional subgroups including Ras, Rho/Rac, Rab, Arf, and Ran, among which Ras appears to be most extensively studied. Ras-related protein 1A (RAP1A) is a member of Ras subgroup and functions as a crucial regulator in T cell response [8]. It is also found to involve in cell dynamics, bacterial infection and osteoblast differentiation [9, 10]. Recently, increasing studies have demonstrated its overexpression may contribute to the malignant progression of numerous human malignancies, including ovarian, prostate, esophageal and brain cancer [11–14]. For example, RAP1A promotes the growth and metastasis of ovarian cancer through epithelial-mesenchymal transition (EMT) via mitogen-activated protein kinase (MAPK) and Notch signaling pathway [11]. In glioblastoma, RAP1A is essential for thrombin-stimulated proliferation of cancer cells, which can be attributed to its activating role in integrin/ERK or B-Raf/ERK signaling [14]. The mechanism investigations also suggest several micoRNAs (such as miR-196a and miR-203) exert their oncogenic or anti-cancer roles through targeting RAP1A [13, 15]. Taken together, these evidences collectively indicate that RAP1A may be a promising biomarker for cancer diagnosis and treatment.

Despite emerging studies supporting the crucial role of RAP1A in tumorigenesis, to our knowledge, its specific clinical significance and biological function in CRC are still unknown. Therefore, in this study, we firstly analyzed its expression and clinical significance in a retrospective cohort study enrolling 144 CRC patients. Then, we performed a series of functional assays in vitro and in vivo to validate its oncogenic role in CRC cells. Finally, we utilized microarray analysis to identify dominant signaling pathways regulated by RAP1A during CRC progression and performed related molecular validations accordingly.

## Materials and methods

### Patient data

144 pairs of CRC tissues and matched adjacent normal tissues were collected from CRC patients who received surgical treatment from February 2009 to September 2016, at Department of General Surgery, Shanghai Jiao Tong University Affiliated Sixth People’s Hospital. None of patients were diagnosed as metastatic CRC. Neither preoperative chemotherapy nor radiotherapy was performed on patients. Tumor stage was classified according to the Tumor-node-metastasis (TNM) staging system (7th edition, American Joint Committee on Cancer). For postoperative chemotherapy, a standardized FOLFOX scheme was recommended to well-tolerated stage III patients and high-risk stage II patients. For evaluating clinical outcome, overall survival (OS) and disease-free survival (DFS) were utilized. OS was calculated as the time from the primary surgery to the date of death or the last follow-up, while DFS was calculated as the time from the primary surgery to the date of the first CRC recurrence or metastasis. The basic clinicopathological features of patients were shown in Table 1.

**Table 1:** Correlations between RAP1A expression and clinicopathological characteristics in CRC patients

Characteristics	Total	RAP1A expression		*P* value
		Low	High	
Gender				
Male	60	29	31	0.844
Female	84	42	42	
Age				
≤ 60 years	59	24	35	0.084
> 60 years	85	47	38	
Tumor location				
Colon	122	62	60	0.392
Rectal	22	9	13	
Tumor size				
≤ 5 cm	97	48	49	0.951
> 5 cm	47	23	24	
Tumor differentiation				
Well/moderate	110	53	57	0.628
Poor	34	18	16	
Tumor invasion				
T1-T2	27	18	9	*0.045*
T3-T4	117	53	64	
Lymph node metastasis				
Absent	70	36	34	0.620
Present	74	35	39	
Serum CEA level				
≤ 5 ng/ml	87	46	41	0.290
> 5 ng/ml	57	25	32	

Italicized values are less than 0.05

### Immunohistochemistry (IHC) and staining evaluation

The IHC was performed on tissue samples as described previously [16]. Briefly, paraffin-embedded tissues were sectioned into 4 μm-thick, dewaxed in xylene (Sinopharm Chemical Reagent Co., Ltd., China) and rehydrated in alcohol with gradient concentrations. To recover antigen reactivity, the sections were heated in citrate buffer (PH 6.0) using microwaving. Then the sections were incubated in 3% H_2_O_2_ to block endogenous peroxidase activity. After three washes with phosphate buffered solution (PBS),the sections were incubated with the following primary antibodies overnight at 4 °C: anti-RAP1A (Abcam, UK, 1:100) and anti-Ki-67 (Santa Cruz Biotechnology, USA, 1:200). Negative controls were prepared by incubating sections with PBS instead of the primary antibodies. Finally, the sections were incubated with the secondary antibody (Abcam, 1:200) for 1 h at 37 °C and protein staining was visualized using Diaminobenzidine tetrahydrochloride (DAB, Invitrogen Life Technologies, USA).

Staining evaluation was performed independently by two researchers who were blind to the information of the sections. For RAP1A, its staining was evaluated based on staining area and staining intensity. Staining area was scored as follows: 0(< 5%), 1 (5–25%), 2 (26–50%), 3 (51–75%), and 4 (76–100%). Staining intensity was scored as follows: 0 (negative); 1 (weak); 2 (medium); and 3 (strong). A final score was calculated by multiplying the both scores together and its cut-off value was determined by receiver operating characteristic (ROC) curve analysis. The ROC curve was constructed based on the staining scores and the OS of patient. The sections scored more or less than its cut-off value were identified as high or low expression cases. For Ki-67, its staining was evaluated only based on the Percentage of Positive cells.

### Cell culture, short hairpin RNAs and plasmid construction

Three human CRC cell lines (HT-29, SW620, HCT116) were purchased from the Type Culture Collection of the Chinese Academy of Sciences (Shanghai, China), while the rest (DLD1, Caco-2 and SW480) and human normal intestinal epithelial cells (HIEC-6) were purchased from the American Type Culture Collection (ATCC, USA). All the culture mediums were supplemented with 10% fetal bovine serum (Gibco) and 100 U/ml penicillin and 100 μg/ml streptomycin.

To stably knockdown RAP1A in vitro, three short hairpin RNAs (shRNA) sequences were designed as follows:5’-GAAGATGTTCCAATGATTT-3’(KD1), 5’-CAACGATAGAAGATTCCTA-3’(KD2), 5’-TCTGACAGTTCAGTTTGTT-3′ (KD3). The negative control was designed as follows: 5’-TTCTCCGAACGTG TCACGT-3′. Then the synthesized DNA oligos containing these sequences were cloned into GV248 vectors (Genechem, China) and verified by DNA sequencing. The lentivirus was synthesized using GV248 vector, pHelper 1.0 and pHelper 2.0 (Genechem), and transfected into HEK-293 T cells using Lipofectamine 3000 (Thermo Fisher Scientific, USA). After incubation for 48 h, the cultured lentiviral supernatant was collected and purified.

The lentivirus infection was performed according to the multiplicity of infection (MOI). To overexpress RAP1A and FOXO3 in CRC cells, the gene sequence in plasmid was constructed using the following NCBI Reference Sequence: NM_001010935.2 (RAP1A); NM_001455.3 (FOXO3). The transfection procedure was performed according to our previous description [17].

### Quantitative real-time reverse transcription PCR (qRT-PCR)

Total RNA was extracted from cells and tissues using Trizol reagent (Invitrogen, USA). Then, reverse-transcription was conducted using M-MLV Reverse Transcriptase (Promega, USA). Subsequently, the polymerase chain reaction was performed using SYBR® Premix Ex TaqTM kit (Takara, Japan) on ABI PRISM 7500 Sequence Detection System (Applied Biosystems, USA). All the procedures were performed following the manufacturer’s instructions. The primer sequences of genes were summarized in Additional file 1: Table S1. The relative mRNA level of gene expression was calculated as ΔCt = Ct_gene_ − Ct_reference_, and the fold change of gene expression was determined by the 2^−ΔΔCt^ method. All the experiments were repeated in triplicate respectively and GAPDH was utilized as an internal control.

### Western blot

The total protein of cells and tissues was extracted using lysis buffer (Genechem, China) supplemented with protease inhibitor (Complete mini, USA). The protein concentration was measured using the bicinchonininc acid (BCA) protein assay kit (Beyotime Biotechnology, China).Then proteins were loaded and separated using 10% sodium dodecyl sulphate-polyacrylamide gel electrophoresis (SDS-PAGE) and transferred onto polyvinylidenedifloride (PVDF) membranes. The membranes were subsequently blocked with 5% non-fat milk for 2 h and incubated with primary antibodies overnight at 4 °C. The primary antibodies used were as follows: anti-RAP1A (1:1000, Abcam, UK), anti-Forkhead box O3 (FOXO3) (1:1000, Cell Signaling Technology, USA), anti-PTEN (1:1000, Abcam), anti-cyclin D1 (CCND1, 1:1000, Abcam) and anti-β-actin (1:5000, Abcam) or anti-GAPDH (1:5000, Santa Cruz Biotechnology, USA). The membranes were incubated with secondary antibody (1:5000, Santa Cruz Biotechnology) for 1 h at room temperature. Finally, the blots were visualized using Chemiluminescence Detection Kit (Thermo Fisher Scientific). The protein expression was quantified using Quantity One software and β-actin or GAPDH served as the internal control.

### TUNEL assay

The paraffin-embedded tissues were cut into sections, dewaxed and rehydrated. The sections were incubated with Proteinase K solution for 30 min. The TUNEL solution was prepared using Enzyme solution and label solution. Then, the sections were incubated with TUNEL solution for 60 min at 37 °C. After three washes with PBS solution, the sections were incubated with DAPI solution (Thermo Fisher Scientific) for 10 min at 37 °C. The staining images was photographed by a fluorescence microscope and the apoptotic cells were scanned using an automatic digital slide scanner (Pannoramic MIDI, 3DHISTECH Ltd., Hungary).

### MTT assay

CRC cells were seeded into 96-well plates (2000/well) with 100 μl fresh culture medium. After overnight incubation, 20 μl MTT reagent (Genview, USA) was added to each well for 4 h incubation at 37 °C.Then the culture medium was removed and 100 μl DMSO reagent (Sinopharm Chemical Reagent, China) was added to dissolve the precipitates. Finally, the optical density (OD) of each well was analyzed by a microplate reader (Biotek, USA) at 490 nm wavelength.

### Apoptosis and cell cycle assay

The cell apoptosis and cell cycle was detected by a flow cytometry (Millipore, USA). Briefly, for the cell apoptosis assay, the CRC cells were washed with D-Hanks buffer and incubated in 200 μl binding buffer containing 10 μlAnnexinV-APC reagent (eBioscience, USA). For the cell cycle analysis, the cells were fixed in 70% ethanol overnight and incubated in PBS buffer containing 50 μg/ml Propidium Iodide and 100 U/ml RNaseA (all from Sigma-Aldrich).

### Clone formation assay

Cells were cultured in a complete medium for 12 days until colonies were formed. Culture medium was changed at regular time intervals. Then the colonies were washed three times with PBS and fixed with 4% paraformaldehyde for 30 min. Finally, the colonies were stained with Giemsa (Chemicon, Japan) reagent for 20 min and washed twice with double distilled water. The number of colonies were counted using a microscopy (Olympus, Japan).

### Xenograft model

Twenty-four athymic male BALB/c nude mice (4–5 weeks old, 15-18 g) were purchased from Shanghai Lab. Animal Research Center and housed in specific-pathogen-free conditions. 3.0 × 10^6^ CRC cells were injected subcutaneously into the left hind flank of each mouse. Tumor volumes were monitored every four days a week by a caliper and calculated according the formula as follows: volume = 0.5 × length×width^2^. Four weeks after the injection, mice were sacrificed and the tumors were harvested. Tumor weights were measured and the harvested tumor samples were reserved in 4% paraformaldehyde (Sangon Biotech, Shanghai, China).

### Microarray analysis

Gene profiling was analyzed using GeneChip™ PrimeView™ Human Gene Expression Array (Affymetrix, USA) according to our previous description [16]. In brief, total RNA was extracted from CRC cells transfected with shRNA and its negative control. Eligible RNA samples were verified using Nanodrop 2000 (Thremo Fisher Scientific, USA) and 2100 Bioanalyzer (Aglient, USA). Then, the samples were transferred to In vitro transcription (IVT) using GeneChip 3’IVT Express Kit (Affymetrix, USA). Subsequently, arrays were hybridized using GeneChip Hybridization Oven 645 (Affymetrix), stained using GeneChip Fluidics Station 450 (Affymetrix) and scanned using the GeneChip Scanner 3000 (Affymetrix). Finally, the microarray data were processed by Ingenuity Pathway Analysis (IPA) online (www.ingenuity.com). A *p* value less than 0.05 indicates significant expression.

### Statistical analysis

The data are presented as mean ± standard deviation (SD). Statistical analysis was performed using SPSS software package version 22.0 (SPSS, USA). The Chi-square test was used to analyze the correlations between RAP1A expression and clinicopathological parameters. Survival curves were constructed using Kaplan Meier method and compared using the log-rank test. The univariate and multivariate analysis were carried out to assess significant prognostic factors by Cox proportional hazards regression model. For cellular assays in vitro and in vivo, statistical significance between two groups was determined by a two-sided Student’s t test. For all analysis, a *p*-value less than 0.05 was considered to be statistically significant.

## Results

### Expression and clinical significance of RAP1A in CRC patients

**Fig. 1:**
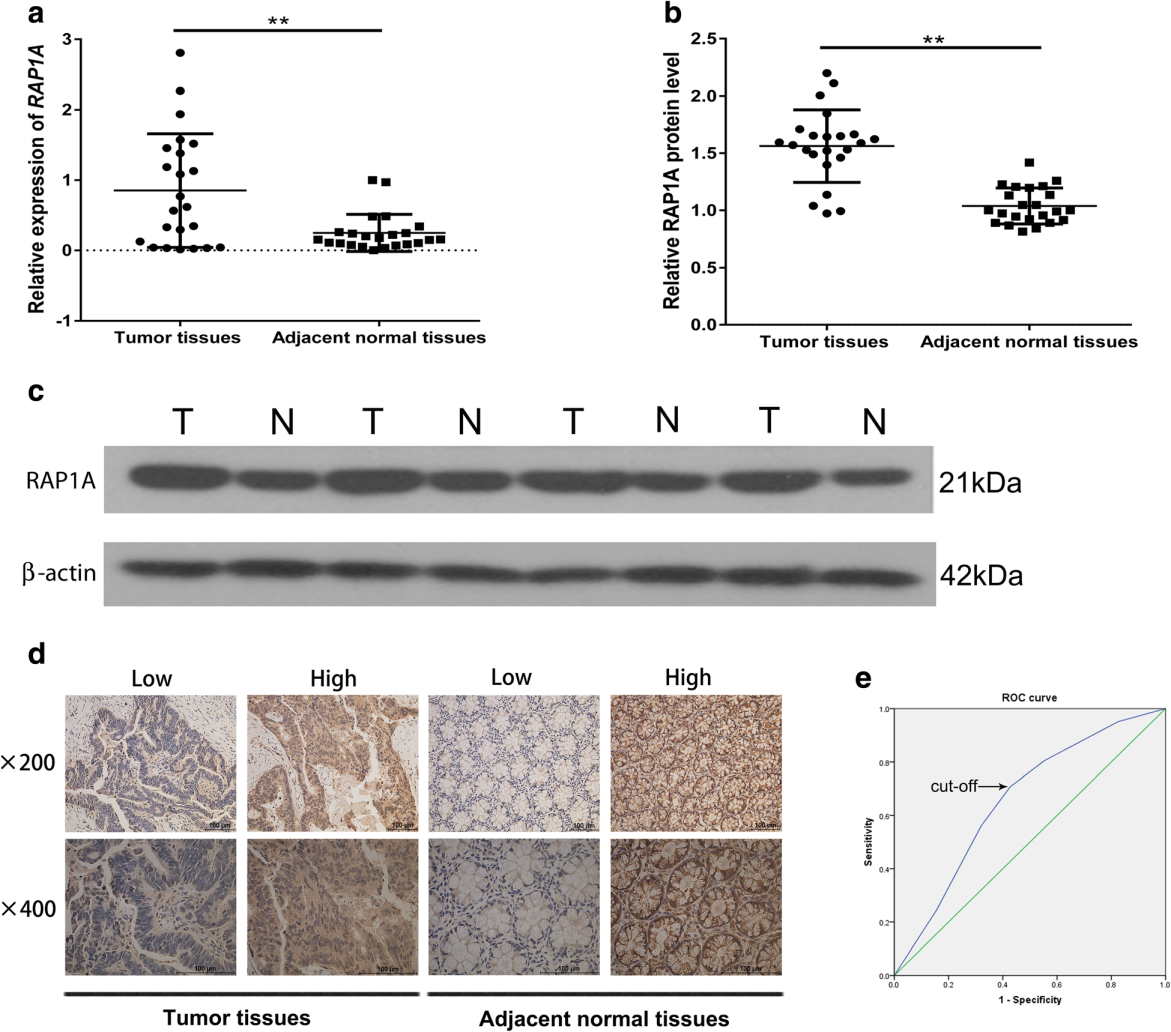
Expression of RAP1A in CRC and matched adjacent normal tissues. **a**: qRT-PCR assay indicates the mRNA level of *RAP1A* gene was higher in CRC tissues than that in matched adjacent normal tissues (*n* = 23, *p* = 0.0015). **b**: Western blot indicates the protein level of RAP1A was higher in CRC tissues than that in matched adjacent normal tissues (n = 23, *p* < 0.0001). **c**: Representative protein bands of RAP1A and β-actin. **d**: Representative immunohistochemical staining images of RAP1A in CRC and matched adjacent normal tissues. **e**: The cut-off value of immunohistochemical staining scores was determined by receiver operating characteristic curve analysis, with sensitivity of 70.7% and specificity of 57.3%. ** means *p* < 0.01

Firstly, using qRT-PCR, we found the mRNA level of *RAP1A* gene was significantly higher in CRC tissues as compared with that in matched adjacent normal tissues (*n* = 23, *p* = 0.0015, Fig. 1a). This finding was then supported by western blot (n = 23, *p* < 0.0001, Fig. 1b and c). For further understanding the clinical significance of RAP1A, IHC was used and the representative images of IHC were shown in Fig. 1d. The ROC analysis demonstrated the cut-off value of staining scores was 3.5. (Fig. 1e). Therefore, we divided the whole cohort into high expression group (*n* = 73) and low expression group (*n* = 71) based on this cut-off value.

As shown in Table 1, we found RAP1A expression was significantly correlated with tumor invasion (*p* = 0.045). No significant correlation was found between RAP1A expression and other clinicopathological parameters including gender (*p* = 0.844), age (*p* = 0.084), tumor location (*p* = 0.392), tumor size (*p* = 0.951), tumor differentiation (*p* = 0.628), lymph node metastasis (*p* = 0.620) and preoperative CEA level (*p* = 0.290).

**Fig. 2:**
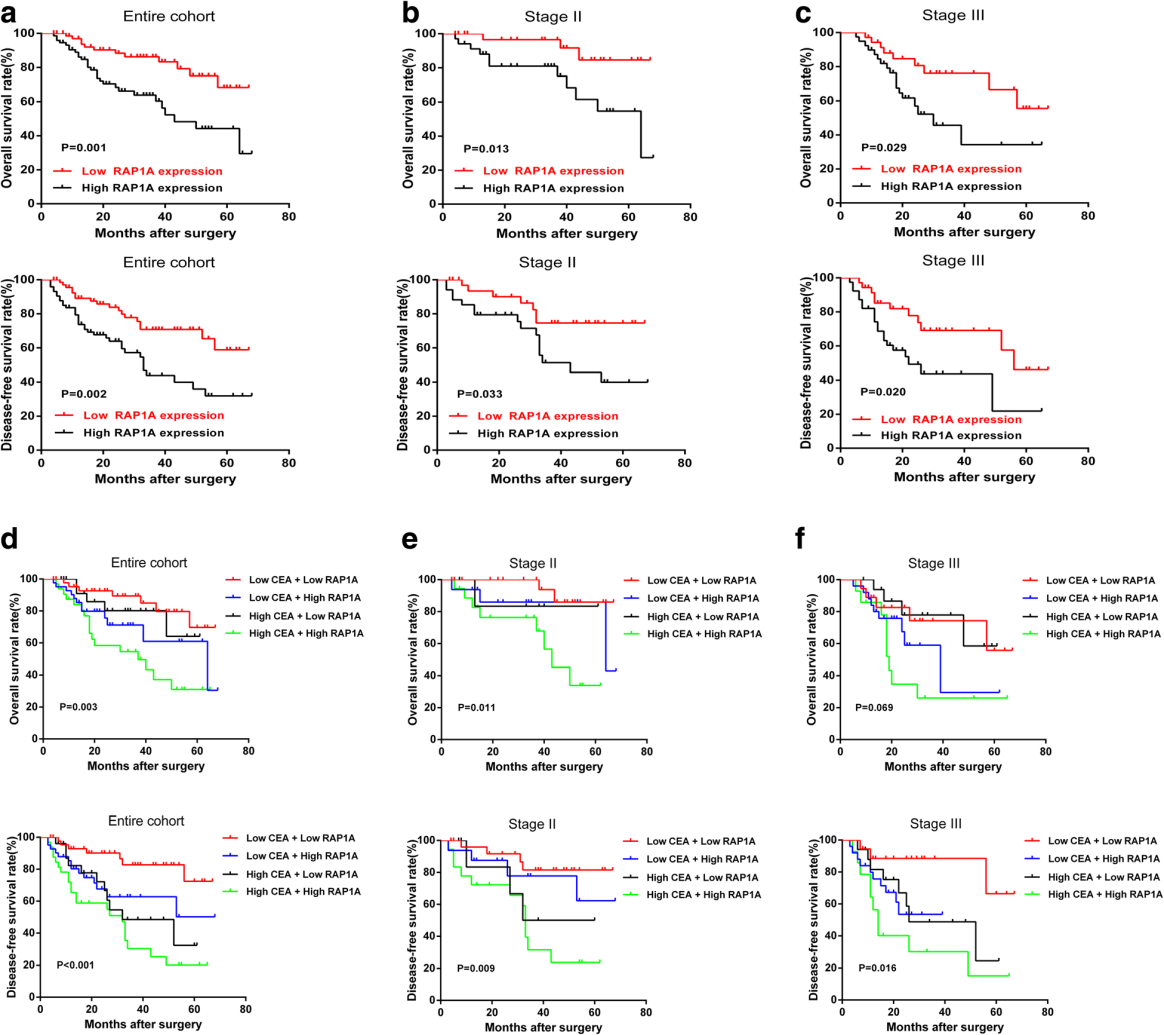
Prognostic significance of RAP1A in CRC patients. **a**: Overall survival (OS) and disease-free survival (DFS) curves of the whole cohort stratified by RAP1A expression. **b**: OS and DFS curves of stage II patients stratified by RAP1A expression. **c**: OS and DFS curves of stage III patients stratified by RAP1A expression. **d**: OS and DFS curves of the whole cohort stratified by RAP1A expression and preoperative serum CEA level. **e**: OS and DFS curves of stage II patients stratified by RAP1A expression and preoperative serum CEA level. **f**: OS and DFS curves of stage III patients stratified by RAP1A expression and preoperative serum CEA level

The prognostic significance of RAP1A was illustrated by Kaplan Meier survival curves. As shown in Fig. 2a, for the whole cohort, high RAP1A expression was associated with worse OS and DFS than low RAP1A expression (*p* = 0.001 and *p* = 0.002, Fig. 2a). In the univariate analysis (shown in Table 2), we found RAP1A expression, lymph node metastasis and preoperative CEA level were significant prognostic factors for both OS and DFS (all *p* < 0.05). In the multivariate analysis (shown in Table 3), we found RAP1A expression and lymph node metastasis were independent prognostic factors for OS, while RAP1A expression, lymph node metastasis and preoperative CEA level were for DFS (all * p* < 0.05). In subgroup analysis based on TNM stage, we observed a significant correlation between RAP1A expression and OS/DFS both in stage II and III patients (all *p* < 0.05, Fig. 2b and c). Since CEA is the most commonly examined tumor marker for CRC patients, we then integrated its preoperative level with RAP1A expression in prognostic analysis. As a result, in the whole cohort, we found patients with both high RAP1A expression and preoperative CEA level had a significantly worse OS and DFS than other patients (all *p* < 0.05, Fig. 2d). Furthermore, we also found this combination could stratify the OS/DFS in stage II (all *p* < 0.05) and DFS in stage III patients (*p* = 0.016).

**Table 2:** Univariate analysis for prognostic factors affecting overall survival and disease-free survival

Variables	OS			DFS		
	HR	95% CI	*P* value	HR	95% CI	*P* value
Gender						
Male vs. Female	0.883	0.477–1.633	0.691	1.029	0.600–1.767	0.916
Age						
≤ 60 years vs. > 60 years	0.755	0.408–1.395	0.369	0.743	0.435–1.269	0.277
Tumor location						
Colon vs. Rectal	1.801	0.856–3.791	0.121	1.870	0.983–3.560	0.057
Tumor size						
≤ 5 cm vs. > 5 cm	0.849	0.425–1.695	0.643	1.454	0.841–2.514	0.180
Tumor differentiation						
Well/moderate vs. Poor	1.071	0.525–2.187	0.850	1.014	0.533–1.929	0.967
Tumor invasion						
T1-T2 vs. T3-T4	1.394	0.585–3.321	0.454	1.610	0.726–3.567	0.241
Lymph node metastasis						
Absent vs. Present	2.298	1.194–4.424	*0.013*	1.759	1.016–3.048	*0.044*
Serum CEA level						
≤ 5 ng/ml vs. > 5 ng/ml	1.926	1.039–3.572	*0.038*	2.635	1.523–4.558	*0.001*
RAP1A expression						
Low vs. High	2.887	1.467–5.681	*0.002*	2.396	1.356–4.233	*0.003*

Italicized values are less than 0.05

**Table 3:** Multivariate analysis for prognostic factors affecting overall survival and disease-free survival

Variables	OS			DFS		
	HR	95% CI	*P* value	HR	95% CI	*P* value
Gender						
Male vs. Female	0.630	0.329–1.207	0.164	0.742	0.418–1.318	0.309
Age						
≤ 60 years vs. > 60 years	1.159	0.590–2.277	0.668	1.058	0.589–1.903	0.850
Tumor location						
Colon vs. Rectal	1.482	0.650–3.382	0.350	1.765	0.862–3.614	0.121
Tumor size						
≤ 5 cm vs. > 5 cm	0.813	0.393–1.684	0.578	1.393	0.791–2.452	0.251
Tumor differentiation						
Well/moderate vs. Poor	1.061	0.500–2.248	0.878	0.942	0.476–1.866	0.865
Tumor invasion						
T1-T2 vs. T3-T4	1.126	0.428–2.960	0.810	1.276	0.535–3.048	0.583
Lymph node metastasis						
Absent vs. Present	2.516	1.254–5.050	*0.009*	1.863	1.037–3.347	*0.037*
Serum CEA level						
≤ 5 ng/ml vs. > 5 ng/ml	1.821	0.964–3.439	0.065	2.412	1.372–4.238	*0.002*
RAP1A expression						
Low vs. High	2.823	1.397–5.707	*0.004*	2.149	1.189–3.882	*0.011*

Italicized values are less than 0.05

### Knockdown of RAP1A inhibits the growth of CRC cells in vitro

**Fig. 3:**
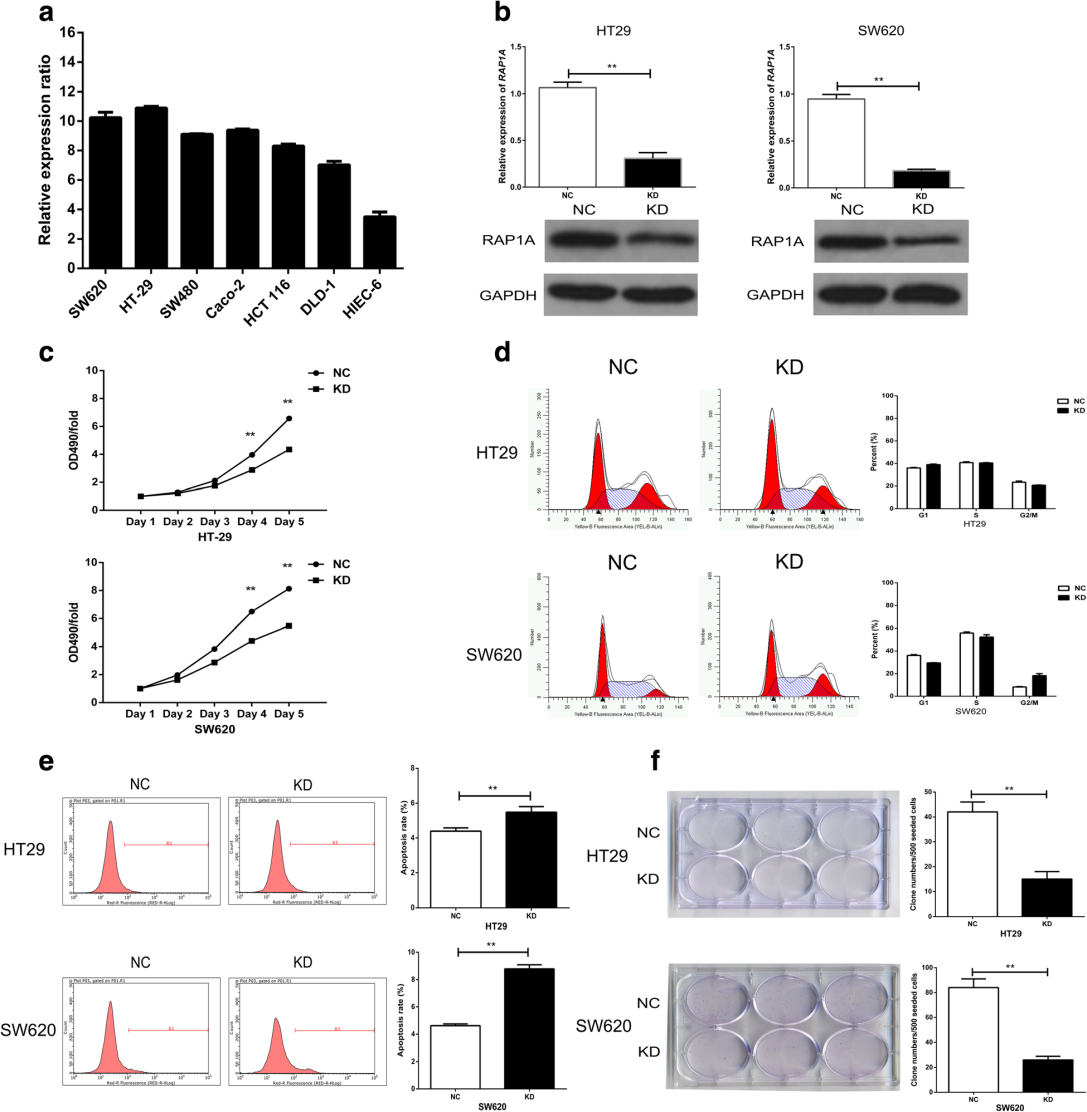
Knockdown of RAP1A inhibits the growth of CRC cells in vitro*.* **a**: qRT-PCR detected the mRNA level of *RAP1A* gene is higher in CRC cell lines as compared with normal intestinal epithelial cells. **b**: qRT-PCR and western blot showed both mRNA and protein expression of RAP1A is lower in knockdown (KD) group than that of negative control (NC) group. **c**: the relative 490 nm absorbance of KD group increases more slowly than that of NC group within five days. **d**: upper: there is no significant difference in cycle progression after RAP1A knockdown in HT-29 cells; lower: RAP1A knockdown decreases G1 phase fraction but increases G2/M phase fraction in SW620 cells. **e**: the apoptosis rates of KD group are higher than that of NC group. **f**: the clone numbers of KD group are less than that of NC group. ** means *p* < 0.01

Firstly, we performed qRT-PCR to detect *RAP1A* expression in six commonly used CRC cell lines and one normal intestinal epithelial cell line. We found it is significantly higher in CRC cells as compared with that in normal control, and is most abundant in HT-29 and SW620 CRC cells (Fig. 3a). Therefore, we used both the cell lines in the following cellular assays. Then, we designed three shRNAs (KD1, KD2 and KD3) to knockdown RAP1A in vitro and validated their efficiency in SW620 cells. As result, we found KD3 had the best knockdown efficiency at mRNA level (shown in Additional file 2: Figure S1). In addition, before functional assays, we proved that KD3 significantly inhibited RAP1A expression in HT-29 and SW620 cells both at mRNA and protein level (Fig. 3b). In the MTT assays, knockdown of RAP1A dramatically suppressed the growth of HT-29 and SW620 cells from the fourth day (all *p* < 0.05, Fig. 3c). The cell cycle analysis demonstrated that knockdown of RAP1A had a unconspicuous impact on the cell cycle progression of HT-29 cells, but it significantly induced arrest in G2/M phase in SW620 cells (Fig. 3d). The apoptosis assay showed knockdown of RAP1A significantly increased the apoptosis rate of HT-29 and SW620 cells (all * p* < 0.05, Fig. 3e). Finally, in the clone formation assays, we observed that knockdown of RAP1A reduced the clone numbers of HT-29 and SW620 cells (all *p* < 0.05, Fig. 3f). Furthermore, we also constructed the specific plasmid to overexpress RAP1A in DLD-1 cell lines (Additional file 3: Figure S2a). As a result, we found RAP1A overexpression increased the proliferation (Additional file 3: Figure S2b), apoptosis resistance (Additional file 3: Figure S2d) and clone formation (Additional file 3: Figure S2e) of DLD-1 cells. No significant changes of cell cycle progression in DLD-1 cells were observed after RAP1A overexpression (Additional file 3: Figure S2c). Taken together, these evidences suggest RAP1A is crucial for the growth of CRC cells in vitro.

### Knockdown of RAP1A inhibits the growth of CRC cells in vivo

**Fig. 4:**
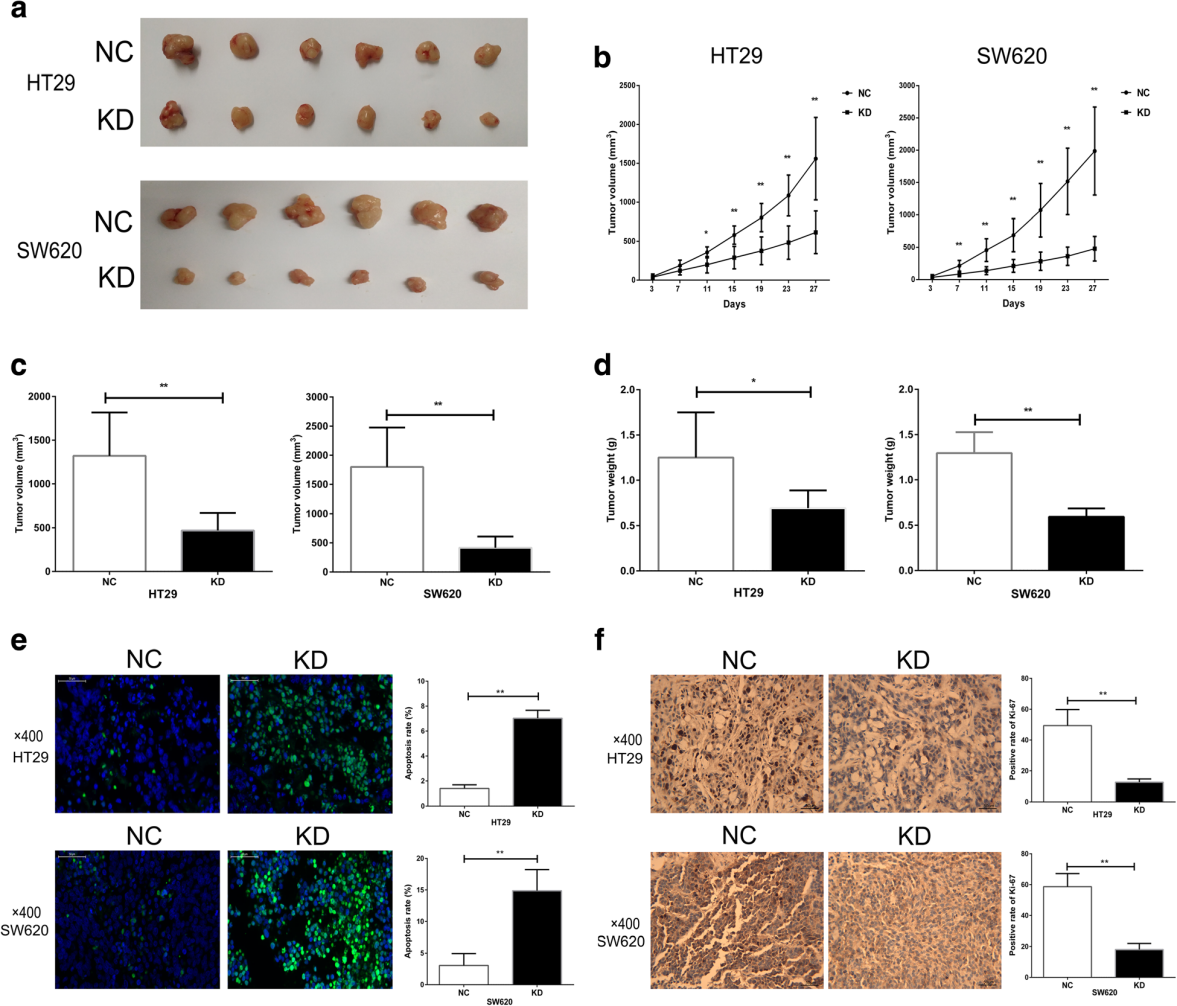
Knockdown of RAP1A inhibits the growth of CRC cells in vivo*.* **a**: Harvested xenografts from nude mice that were subcutaneously injected with SW620 and HT-29 cells. **b**: the increase of tumor volume in knockdown (KD) group is slower than that in negative control (NC) group. **c**: the final tumor volumes of KD group are less than that of NC group. **d**: the final tumor weights of KD group are less than that of NC group. **e**: the rates of apoptotic tumor cells in the harvested xenografts are higher in KD group than that in NC group. **f**: the rates of Ki-67 positive tumor cells in the harvested xenografts are lower in KD group than that in NC group. * means *p* < 0.05. ** means *p* < 0.01

The xenograft model was utilized to investigate the impact of RAP1A on the growth of CRC cells in vivo. The images of harvested tumors were shown in Fig. 4a and we found the sizes of tumors in KD group were obviously smaller than those in NC group. The growth curve analysis suggested the xenograft growth was significantly inhibited after RAP1A knockdown in both HT-29 and SW620 cells (Fig. 4b). Furthermore, both the final volumes and weights of harvested tumors in KD group were significantly less than those in NC group (all *p* < 0.05, Fig. 4c and d). Finally, we detected apoptosis rate and Ki-67 expression in the harvested tumors and found RAP1A knockdown significantly increased the apoptosis rate of CRC cells and reduced the percentage of Ki-67 positive CRC cells (all *p* < 0.05, Fig. 4e and f).

### RAP1A promotes CRC growth through regulating PTEN/FOXO3/CCND1 signaling pathways

**Fig. 5:**
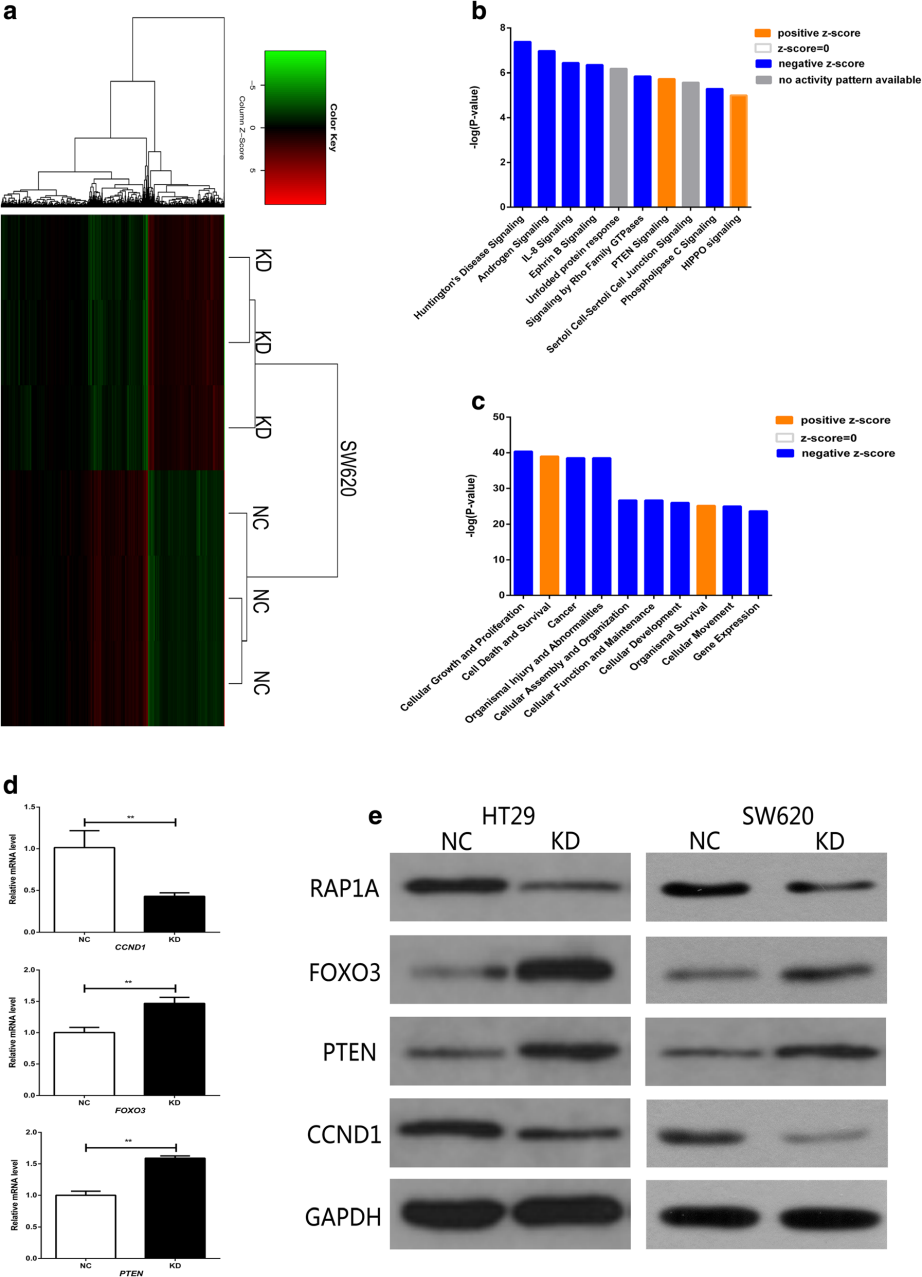
RAP1A promotes CRC growth through regulating PTEN/FOXO3/ CCND1 signaling pathway. **a**: The heat map depicts significantly expressed genes affected by RAP1A knockdown. **b**: Canonical pathway analysis indicates the top ten pathways enriched of significantly expressed genes. **c**: Disease and function analysis indicates the top ten biological functions enriched of significantly expressed genes. **d**: the mRNA expression of *PTEN* and *FOXO3* gene is increased while that of *CCND1* gene is decreased in knockdown (KD) group as compared with negative control (NC) group in SW620 cells. **e**: the protein expression of PTEN and FOXO3 is increased while that of CCND1 is decreased in KD group as compared with NC group in SW620 and HT-29 cells. ** means *p* < 0.01

The microarray analysis was performed to investigate the specific molecular mechanisms regulated by RAP1A in CRC development. Firstly, we examined the significantly expressed genes between SW620 cells transfected with shRNA and NC. As a result, compared with NC group, a total of 824 upregulated genes and 1611 downregulated genes were found in RAP1A-knockdown CRC cells (Fig. 5a). In the canonical pathway analysis (Fig. 5b, Additional file 4: Figure S3 and Additional file 5: Table S2), we found these genes were enriched in some cancer-related signaling pathways such as IL-8 and PTEN signaling pathways, which were described in more details in Additional file 6: Figure S4 and Additional file 7: Figure S5 based on microarray data and literatures. In addition, we utilized Z-score to predict its activation or inactivation pattern in RAP1A-knockdown CRC cells. We found IL-8 signaling pathway was significantly inactivated (Z-score = − 4.867), while it is opposite for PTEN signaling pathway (Z-score = 1.671). In the disease and function analysis (Fig. 5c, Additional file 8: Figure S6), we found these genes were enriched in cellular growth related functions such as cellular growth and proliferation, with its Z-score of − 2.292. This result implies RAP1A knockdown may inhibits CRC growth through regulating some cellular growth related genes. Then, based on the above preliminary analysis and previous literatures, we selected 30 representative genes for qRT-PCR validations in SW620 cells (Fig. 5d and Additional file 9: Figure S7). We found RAP1A knockdown significantly upregulated PTEN and FOXO3, but downregulated CCND1 at mRNA level. Therefore, we speculated RAP1A may drive CRC growth through regulating PTEN/FOXO3/CCND1 signaling pathways. Finally, western blot confirmed our speculation at protein level in both SW620 and HT-29 cells (Fig. 5e).

### FOXO3 overexpression partly mimics the inhibitory role of RAP1A knockdown in CRC growth

**Fig. 6:**
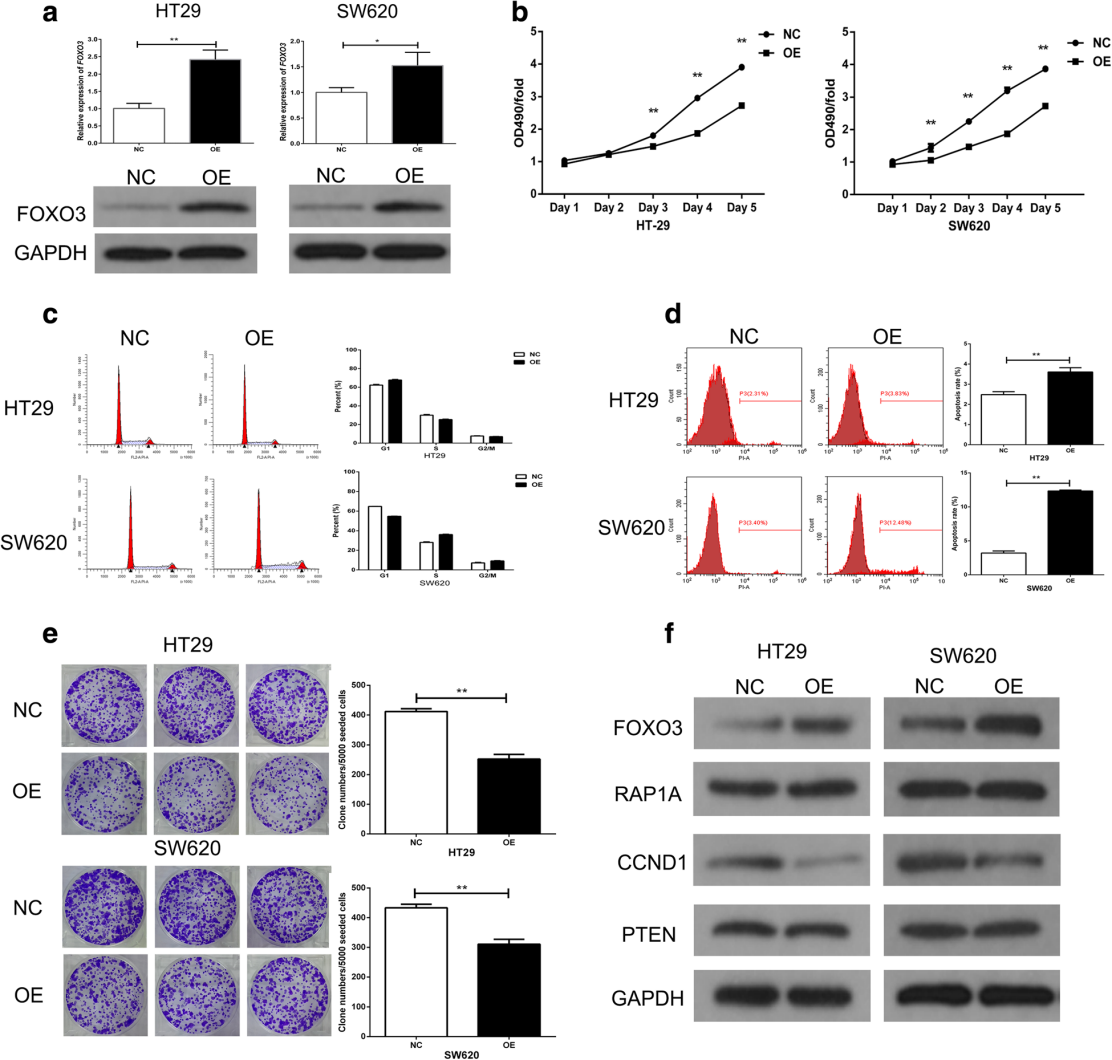
FOXO3 overexpression partly mimics the inhibitory role of RAP1A knockdown in CRC growth. **a**: FOXO3 expression is higher in overexpression (OE) group than that in negative control (NC) group. **b**: the relative 490 nm absorbance of OE group increases more slowly than that of NC group within five days. **c**: upper: FOXO3 overexpression increases G1 phase fraction but decreases S phase fraction in HT-29 cells; lower: FOXO3 overexpression decreases G1 phase fraction but increases G2/M and S phase fraction in SW620 cells. **d**: the apoptosis rates of OE group are higher than that of NC group. **e**: the clone numbers of OE group are less than that of NC group. **f**: the protein expression of CCND1 is increased while no changes of RAP1A and PTEN expression are observed in OE group as compared with NC group. * means *p* < 0.05. ** means *p* < 0.01

To validate whether FOXO3 is a crucial functional downstream of RAP1A, we constructed the specific plasmid to overexpress FOXO3 expression in CRC cells. The qRT-PCR and western blot indicated FOXO3 expression was significantly upregulated after transfecting the plasmid into CRC cells (Fig. 6a). In the MTT assay, we observed FOXO3 overexpression dramatically inhibited the proliferation of CRC cells (Fig. 6b). In addition, using flow cytometry, we also observed FOXO3 overexpression induced the cell cycle arrest and apoptosis of CRC cells (Fig. 6c and d). In the clone formation assay, FOXO3 overexpression decreased the clone number of CRC cells (Fig. 6e). Finally, we used western blot to detect the expression of related proteins in PTEN signaling pathway in CRC cells after FOXO3 overexpression (Fig. 6f). As a result, we found FOXO3 overexpression could inhibit CCND1 expression, but had no impact on the expression of RAP1A and PTEN.

## Discussion

The members of RAS superfamily have been identified as crucial biomarkers for the diagnosis and treatment of numerous human malignancies [18–20]. For example, Ras Superfamily Protein Rap2B functions as a target of p53 to influence tumorigenesis through regulating autophagy [21]. In cervical cancer,

knockdown of Rap2B inhibits the growth, migration and invasion of cancer cells through ERK1/2 signaling pathway [22]. Rab31, another member of Ras superfamily, was reported to promote the malignant progression of glioblastoma and cervical cancer through inducing epithelial-mesenchymal transition [23]. High Rab31 level in tumor tissues was associated with worse 5-year DFS in patients with breast cancer [24]. Similar with these members, RAP1A has recently been found to involve in the development of various cancers, however its specific role in CRC remains poorly investigated.

In this study, we firstly compared RAP1A expression between CRC and adjacent normal tissues. Our result indicated that RAP1A was abnormally overexpressed in CRC tissues at both mRNA and protein level as compared with adjacent normal tissues. To further clarify the clinical correlations of RAP1A, we subsequently examined RAP1A level using IHC in a retrospective study cohort. We found RAP1A level was significantly correlated with T stage, which is partly in accordance with a recent study regarding its clinical significance in oral cavity squamous cell carcinoma [25]. This finding implies that RAP1A may participate in the malignant progression of CRC. Based on the survival curve model, we further observed that patients with high RAP1A expression had a significantly worse OS and DFS than those with low RAP1A expression. The univariate and multivariate analysis also revealed it was an independent prognostic factor for CRC patients. Both the results support RAP1A has the potential to serve as a helpful biomarker in prognostic evaluation.

Previous studies have suggested an accurate prognostic stratification in patients within the same tumor stage is challenging for oncologists because of high tumor heterogeneity in individuals [26–28]. In this regard, traditional TNM staging system may be insufficient for current prognostic evaluation and novel biomarkers are needed. In our subgroup analysis, we found RAP1A expression in primary CRC tissues could stratify the clinical outcome of both stage II and III patients, suggesting RAP1A as a promising prognostic indicator for identifying risk population within the same TNM stage. Since serum CEA is the most commonly detected parameter for CRC patients and numerous studies have proved the prognostic significance of its preoperative level, we next combined RAP1A expression with preoperative CEA level in prognostic evaluation [29–31]. As a result, we found high operative CEA level combined with high RAP1A expression was significantly associated with worse clinical outcome in CRC patients as compared with other phenotypes, and this association remains to be statistically significant in the subgroup analysis, except for OS in stage III patients. Based on these observations, we speculated that RAP1A has the potential to be integrated into traditional clinical system to form a more effective model for prognostic prediction. Finally, it should be mentioned that our study failed to detect the circulating level of RAP1A in CRC patients because of limited clinical resources. Therefore, whether it could still be a useful biomarker and combined with CEA in non-invasive diagnosis needs further investigation in future.

Then, we performed functional assays to investigate the biological role of RAP1A in CRC development. As a result, we found knockdown of RAP1A inhibits the proliferative, anti-apoptosis and clone formation ability of CRC cells in vitro, while the opposite was for RAP1A overexpression. In addition, knockdown of RAP1A induced cell cycle arrest of CRC cells. In vivo, knockdown of RAP1A inhibits the growth of CRC xenografts, as subsequently confirmed by decreased Ki-67 positive cells and increased apoptotic rate. These results strongly support that RAP1A plays a crucial promoting role in CRC growth. In accordance with our results, researchers found RAP1A also promotes the malignant proliferation of other tumors such as brain, prostate and lung cancer [14, 15, 32]. In addition to its role in tumor growth, previous studies have proved it also contributes to the invasion and metastasis of cancer cells [11, 33, 34]. Taken together, these evidences collectively suggest that RAP1A is a key oncoprotein in tumor development and therefore can serve as a potential therapeutical target for cancer patients.

Finally, to preliminarily clarify the molecular mechanism regulated by RAP1A in CRC development, we utilized microarray analysis to identify significantly expressed genes affected by RAP1A in CRC cells. Through Ingenuity Pathway Analysis, we found these genes were significantly enriched in some cell growth related signaling pathways such as IL-8 and PTEN [35, 36]. Then, using qRT-PCR and western blot, we determined that knockdown of RAP1A activates the PTEN signaling pathway, as characterized by upregulated PTEN and FOXO3 expression, and downregulated CCND1 expression. PTEN is a well-established tumor suppressor and increasing studies have suggested it inhibits tumor growth through upregulating FOXO family members such as FOXO3 and FOXO4 [37, 38]. In CRC, a recent study have demonstrated that PTEN/FOXO3 signaling cascade induces the growth of CRC cells through regulating cell-cycle related proteins [39]. CCND1, also known as cyclin D1, is abnormally overexpressed in numerous cancers and promotes uncontrolled tumor growth through influencing cell-cycle progression [40]. More importantly, some previous studies have indicated that CCDN1 serves as a functional downstream of FOXO3 in cancer cells [41–43]. Based on these evidences, we speculated that RAP1A inactivated PTEN/FOXO3 signaling and then upregulated CCND1 to promote CRC growth. For validating our speculation, we overexpressed FOXO3 expression in CRC cells and found it could mimic the inhibitory effect of RAP1A knockdown in CRC growth in vitro. In addition, FOXO3 overexpression significantly inhibited CCND1 expression, but had no impact on RAP1A and PTEN expression. Therefore, we concluded that RAP1A promotes CRC growth through regulating PTEN/FOXO3/CCND1 signaling pathway.

## Conclusions

Our study demonstrates that high RAP1A expression is an independent unfavourable prognostic biomarker for CRC patients. In addition, related cellular assays indicate that RAP1A promotes the growth of CRC cells through regulating PTEN/FOXO3/CCND1 signaling pathway. These findings collectively provide a novel insight into the oncogenic role of RAP1A. Future work should be emphasized on its actual clinical value based on large samples as well as other potential oncogenic roles in CRC development.

## Additional files

Additional file 1: **Table S1.** Primer sequences for the quantitative polymerase chain reaction. (DOC 76 kb)

Additional file 2: **Figure S1.** Upper: The fluorescence microscopy detects the transfection efficiency of each shRNA (KD1, KD2 and KD3). Lower: qRT-PCR assay demonstrates the mRNA level of *RAP1A* gene is lowest in SW620 cells transfected with KD3 shRNA. (TIF 19639 kb)

Additional file 3: **Figure S2.** RAP1A overexpression promotes the growth of DLD-1 CRC cells in vitro. a: RAP1A expression is higher in overexpression (OE) group than that in negative control (NC) group. b: the relative 490 nm absorbance of OE group increases more quickly than that of NC group within five days. c: RAP1A overexpression has no significant impact on the phase fraction in DLD-1 cells. d: the apoptosis rates of OE group are lower than that of NC group. e: the clone numbers of OE group are more than that of NC group. (TIF 4391 kb)

Additional file 4: **Figure S3.** Z-scores of top ten canonical pathways (TIF 322 kb)

Additional file 5: **Table S2.** The top ten significantly changed Canonical Pathway based on microarray analysis. (DOC 41 kb)

Additional file 6: **Figure S4.** Ingenuity Pathway Analysis depicts IL-8 signaling pathways based on microarray data and available literatures (TIF 515 kb)

Additional file 7: **Figure S5.** Ingenuity Pathway Analysis depicts PTEN signaling pathways based on microarray data and available literatures (TIF 1204 kb)

Additional file 8: **Figure S6.** Z-scores of top ten enriched diseases and functions (TIF 305 kb)

Additional file 9: **Figure S7.** qRT-PCR detects the expression of 27 significantly expressed genes in SW620 cells after RAP1A knockdown. (TIF 3186 kb)

## Abbreviations

CCND1: Cyclin D1
CRC: Colorectal cancer
DFS: Disease-free survival
FOXO3: Forkhead box O3
IHC: Immunohistochemistry
OS: Overall survival
PTEN: Phosphatase and tensin homolog
qRT-PCR: Quantitative real-time reverse transcription PCR
RAP1A: Ras-related protein 1A
TNM: Tumor-node-metastasis
