# Supplementary material for: High expression of Ras-related protein 1A promotes an aggressive phenotype in colorectal cancer via PTEN/FOXO3/CCND1 pathway
Source: J Exp Clin Cancer Res. 2018 Jul 31;37:178. doi: 10.1186/s13046-018-0827-y (PMC6069867; doi:10.1186/s13046-018-0827-y)
Supplement: Supplementary file 1 — Table S1. Primer sequences for the quantitative polymerase chain reaction. (DOC 76 kb) [file 13046_2018_827_MOESM1_ESM.doc]

**Table S1.** Primer sequences for the quantitative polymerase chain reaction.

| Gene | Orientation | Sequence (5'‑3') |
| --- | --- | --- |
| JUN | Forward | CGCCAAGAACTCGGACCTC |
|  | Reverse | CCTCCTGCTCATCTGTCACG |
| TP53 | Forward | CCTCCTCAGCATCTTATCC |
|  | Reverse | ACAAACACGCACCTCAAA |
| AKT1 | Forward | TCTTTGCCGGTATCGTGT |
|  | Reverse | TGTCATCTTGGTCAGGTGGT |
| BAX | Forward | TGCTTCAGGGTTTCATCCA |
|  | Reverse | GGCCTTGAGCACCAGTTT |
| BCL2 | Forward | ACTTCGCCGAGATGTCC |
|  | Reverse | ATGACCCCACCGAACTC |
| BCL2L1 | Forward | CTGAATCGGAGATGGAGACC |
|  | Reverse | GAGCTGGGATGTCAGGTCA |
| CCND1 | Forward | GGTGGCAAGAGTGTGGAG |
|  | Reverse | CCTGGAAGTCAACGGTAGC |
| CDKN1A | Forward | TAGGCGGTTGAATGAGAGG |
|  | Reverse | GTGACAGCGATGGGAAGG |
| EGFR | Forward | GGTGACCGTTTGGGAGTT |
|  | Reverse | CCTGAATGACAAGGTAGCG |
| IL-8（CXCL8） | Forward | TGGCAGCCTTCCTGATTT |
|  | Reverse | AACCCTCTGCACCCAGTT |
| MMP9 | Forward | GCACCACCACAACATCAC |
|  | Reverse | ACCACAACTCGTCATCGTC |
| PTEN | Forward | TGGATTCGACTTAGACTTGACCT |
|  | Reverse | TTTGGCGGTGTCATAATGTCTT |
| VEGFA | Forward | AACTTTCTGCTGTCTTGGGT |
|  | Reverse | TCTCGATTGGATGGCAGTA |
| CCND3 | Forward | ACCTGGCTGCTGTGATTGC |
|  | Reverse | GATCATGGATGGCGGGTAC |
| E-cadherin | Forward | AACGCATTGCCACATACA |
|  | Reverse | CGGGCTTGTTGTCATTC |
| EIF4EBP1 | Forward | GATCTGCCCACCATTCCG |
|  | Reverse | CCGCCCGCTTATCTTCTG |
| IGFBP3 | Forward | GCACAGCACCCAGACTTC |
|  | Reverse | ATCCACGCCCTTGTTTCA |
| FOXO4 | Forward | CCGGAGAAGCGACTGACAC |
|  | Reverse | CGGATCGAGTTCTTCCATCCTG |
| IGFBP1 | Forward | TCAGTACCTATGATGGCTCG |
|  | Reverse | TATCTGGCAGTTGGGGTC |
| FOXO3 | Forward | ACCTGATACCTGTTACCAAAGC |
|  | Reverse | CAGAGGAAGCAATAAAGGAAACC |
| PIK3CA | Forward | TGAAGCACCTGAATAGGCAAGTCG |
|  | Reverse | AGAAAGCCCTGTAGAGCATCCATG |
| FOXO1 | Forward | GAGTGGATGGTCAAGAGCGT |
|  | Reverse | TTCCTTCATTCTGCACACGA |
| CXCR1 | Forward | TTTCCGCCAGGCTTACCAT |
|  | Reverse | ACACCATCCGCCATTTTGC |
| MMP7 | Forward | GGGGACTCCTACCCATTTG |
|  | Reverse | TCCAGCGTTCATCCTCATC |
| MMP2 | Forward | GATACCCCTTTGACGGTAAGGA |
|  | Reverse | CCTTCTCCCAAGGTCCATAGC |
| [RPS6KB1](https://www.ncbi.nlm.nih.gov/gene/6198) | Forward | ATTTATTGGCAGCCCACGAAC |
|  | Reverse | GATGCTTCCCCACTCATTGTC |
| BMPR2 | Forward | CAGAATCAAGAACGGCTATGTG |
|  | Reverse | GGATCTCCAATGTGAGACCAAC |
| MTOR | Forward | TCCGACCTTCTGCCTTCAC |
|  | Reverse | ATTGCCTTCTGCCTCTTATGG |
| CXCR2 | Forward | CTGCCTGTCTTACTTTTCC |
|  | Reverse | CAGTTTGCTGTATTGTTGC |
| MIR124-3 | Forward | GGCCCCTCTGCGTGTTCA |
|  | Reverse | TTCACCGCGTGCCTTAA |
| RAP1A | Forward | CGTGAGTACAAGCTAGTGGTCC |
|  | Reverse | CCAGGATTTCGAGCATACACTG |
| GAPDH | Forward | TGACTTCAACAGCGACACCCA |
|  | Reverse | CACCCTGTTGCTGTAGCCAAA |

Abbreviation：

JUN: Jun proto-oncogene; TP53，Tumor protein p53; AKT: AKT serine/threonine kinase 1; BAX: BCL2 associated X; BCL2：BCL2; BCL2L1: BCL2 like 1; CCND1: Cyclin D1; CDKN1A: Cyclin dependent kinase inhibitor 1A; EGFR: Epidermal growth factor receptor; CXCL8: C-X-C motif chemokine ligand 8; MMP9: Matrix metallopeptidase 9; PTEN: Phosphatase and tensin homolog; VEGFA: Vascular endothelial growth factor A; CCND3: Cyclin D3; CDH1(E- cadherin): Cadherin 1; EIF4EBP1: Eukaryotic translation initiation factor 4E binding protein 1; IGFBP3: Insulin like growth factor binding protein 3; FOXO4: Forkhead box O4; IGFBP1: Insulin like growth factor binding protein 1; FOXO3: Forkhead box O3; PIK3CA: Phosphatidylinositol-4, 5-bisphosphate 3-kinase catalytic subunit alpha; FOXO1: Forkhead box O1; CXCR1: C-X-C motif chemokine receptor 1; MMP7: Matrix metallopeptidase 7; MMP2: Matrix metallopeptidase 2; [RPS6KB1](https://www.ncbi.nlm.nih.gov/gene/6198): Ribosomal protein S6 kinaseB1; BMPR2: Bone morphogenetic protein receptor type 2; MTOR: Mechanistic target of rapamycin kinase; CXCR2: C-X-C motif chemokine receptor 2; MIR124-3: MicroRNA 124-3; RAP1A: Ras-related protein 1A. GAPDH: glyceraldehyde-3-phosphate dehydrogenase.
