# Supplementary figures and images for: High expression of Ras-related protein 1A promotes an aggressive phenotype in colorectal cancer via PTEN/FOXO3/CCND1 pathway
Source: J Exp Clin Cancer Res. 2018 Jul 31;37:178. doi: 10.1186/s13046-018-0827-y (PMC6069867; doi:10.1186/s13046-018-0827-y)

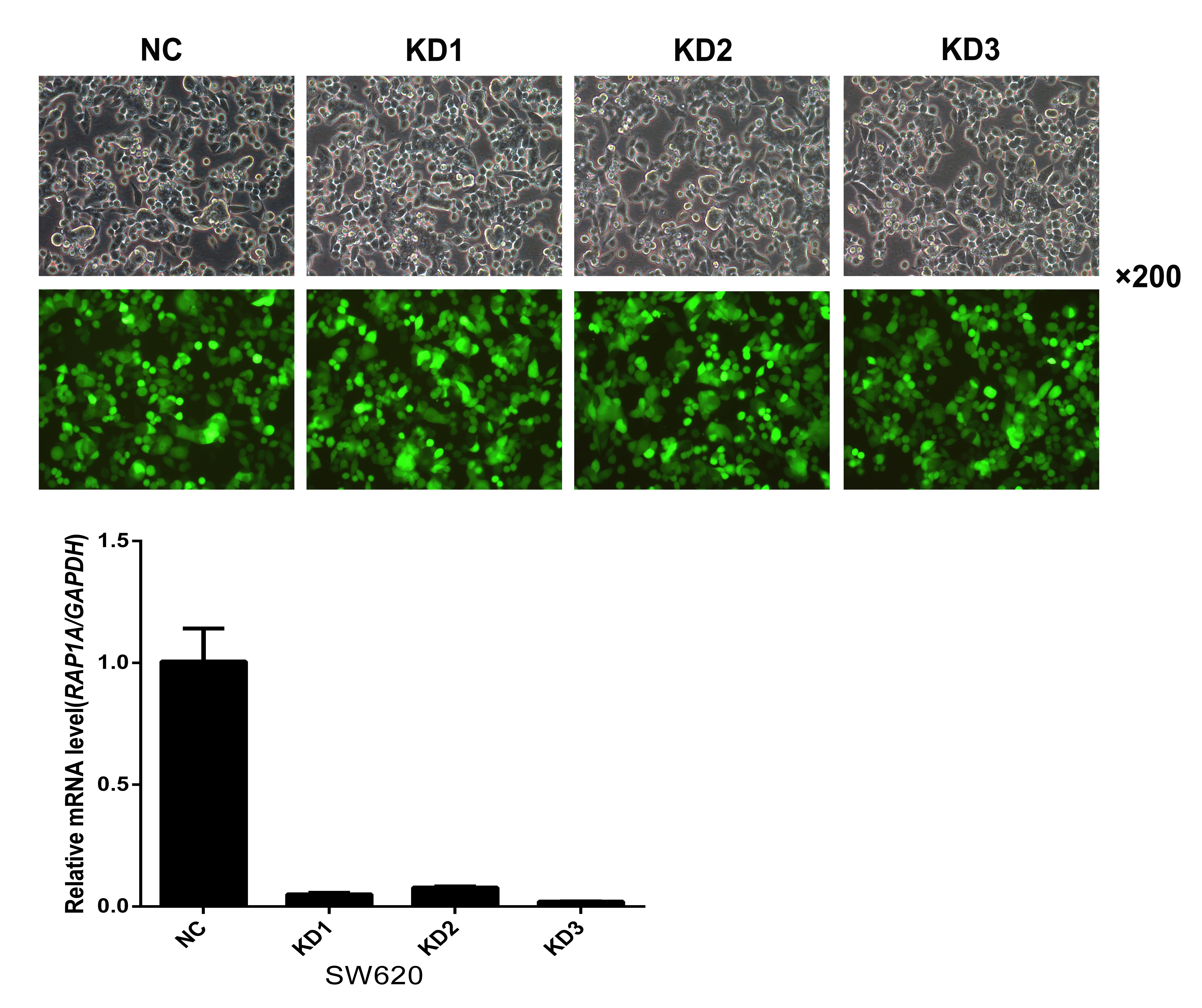

Supplement: Supplementary file 2 — Figure S1. Upper: The fluorescence microscopy detects the transfection efficiency of each shRNA (KD1, KD2 and KD3). Lower: qRT-PCR assay demonstrates the mRNA level of RAP1A gene is lowest in SW620 cells transfected with KD3 shRNA. (TIF 19639 kb) [file 13046_2018_827_MOESM2_ESM.tif]

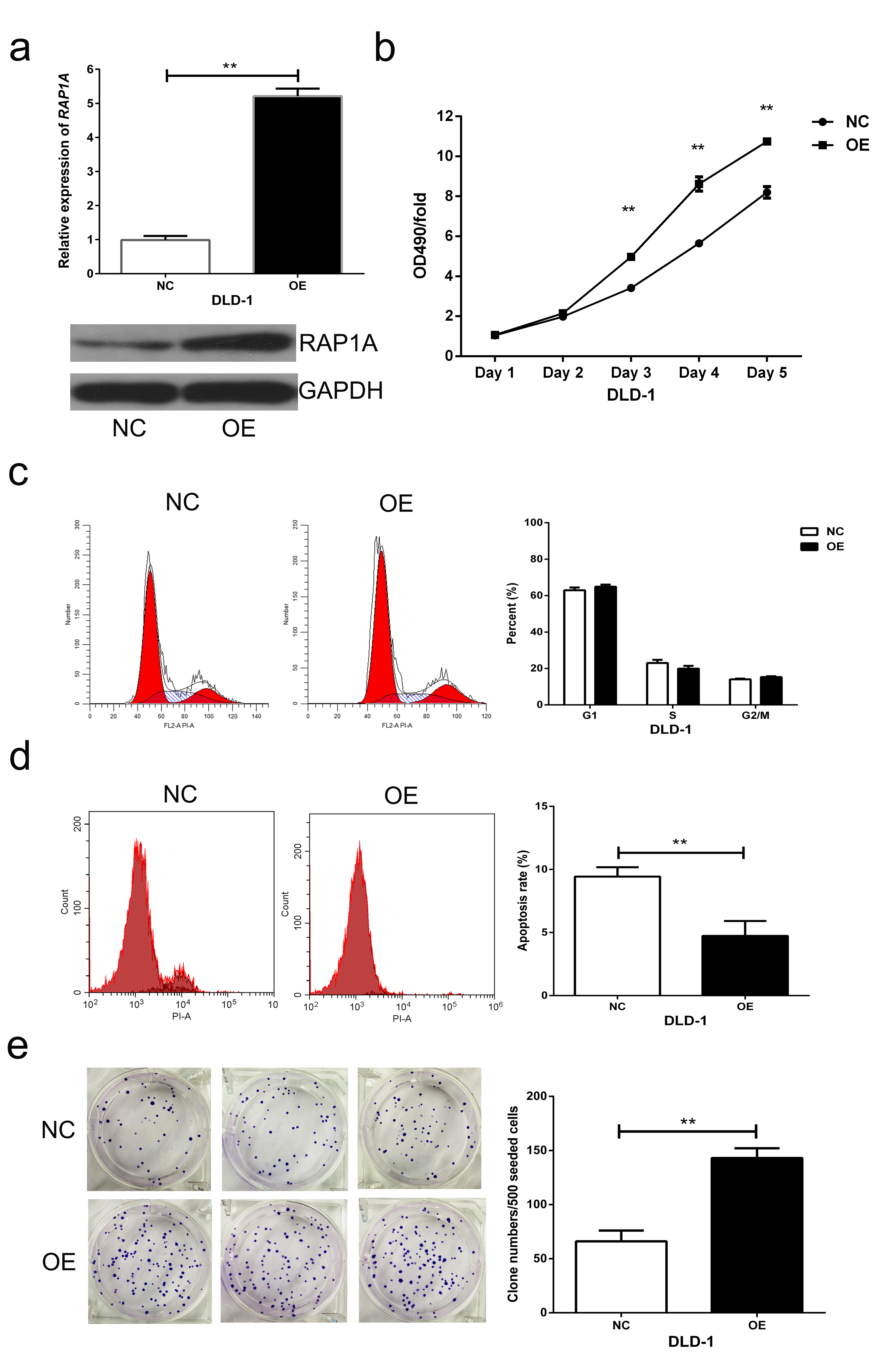

Supplement: Supplementary file 3 — Figure S2. RAP1A overexpression promotes the growth of DLD-1 CRC cells in vitro. a: RAP1A expression is higher in overexpression (OE) group than that in negative control (NC) group. b: the relative 490 nm absorbance of OE group increases more quickly than that of NC group within five days. c: RAP1A overexpression has no significant impact on the phase fraction in DLD-1 cells. d: the apoptosis rates of OE group are lower than that of NC group. e: the clone numbers of OE group are more than that of NC group. (TIF 4391 kb) [file 13046_2018_827_MOESM3_ESM.tif]

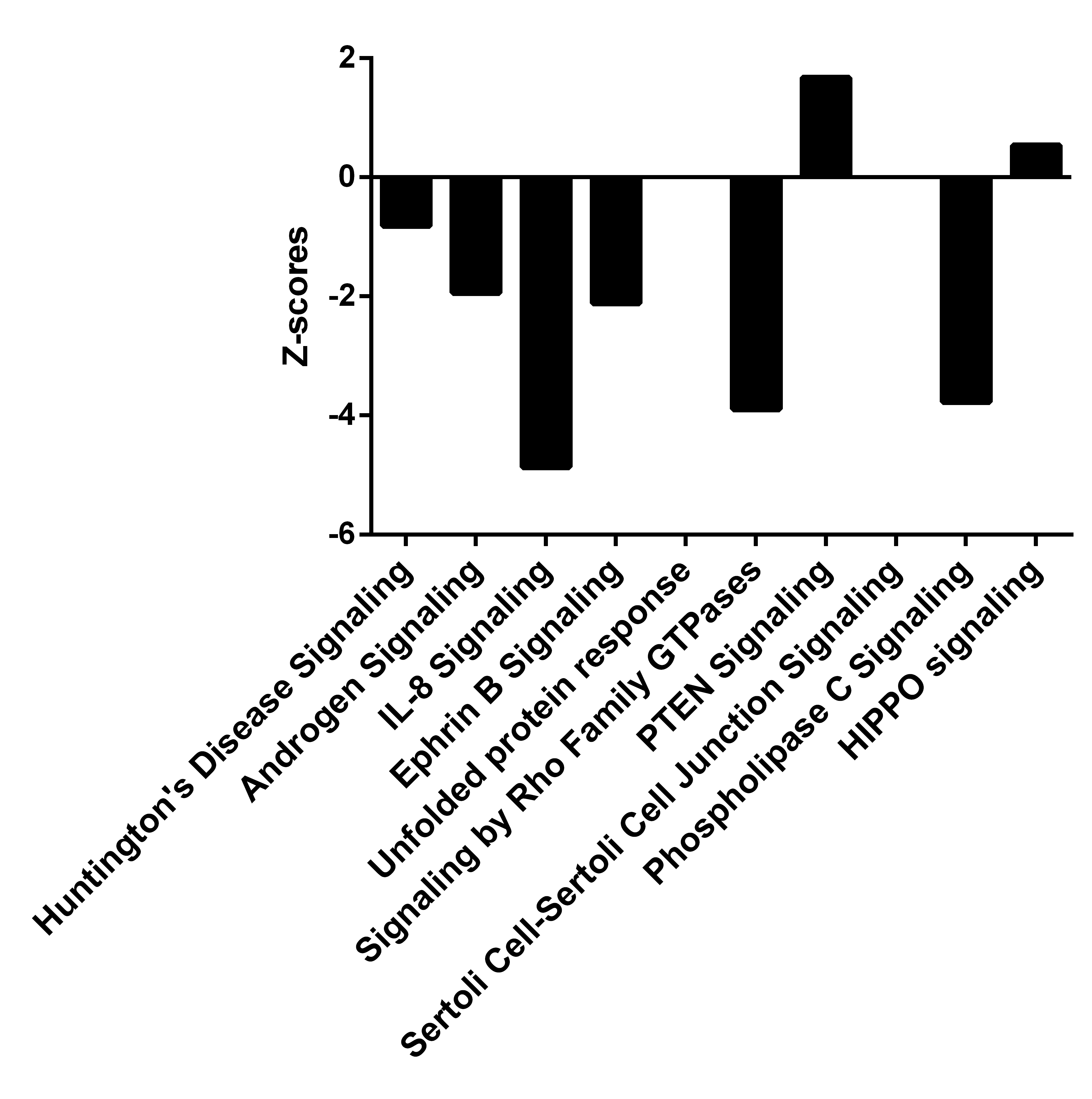

Supplement: Supplementary file 4 — Figure S3. Z-scores of top ten canonical pathways (TIF 322 kb) [file 13046_2018_827_MOESM4_ESM.tif]

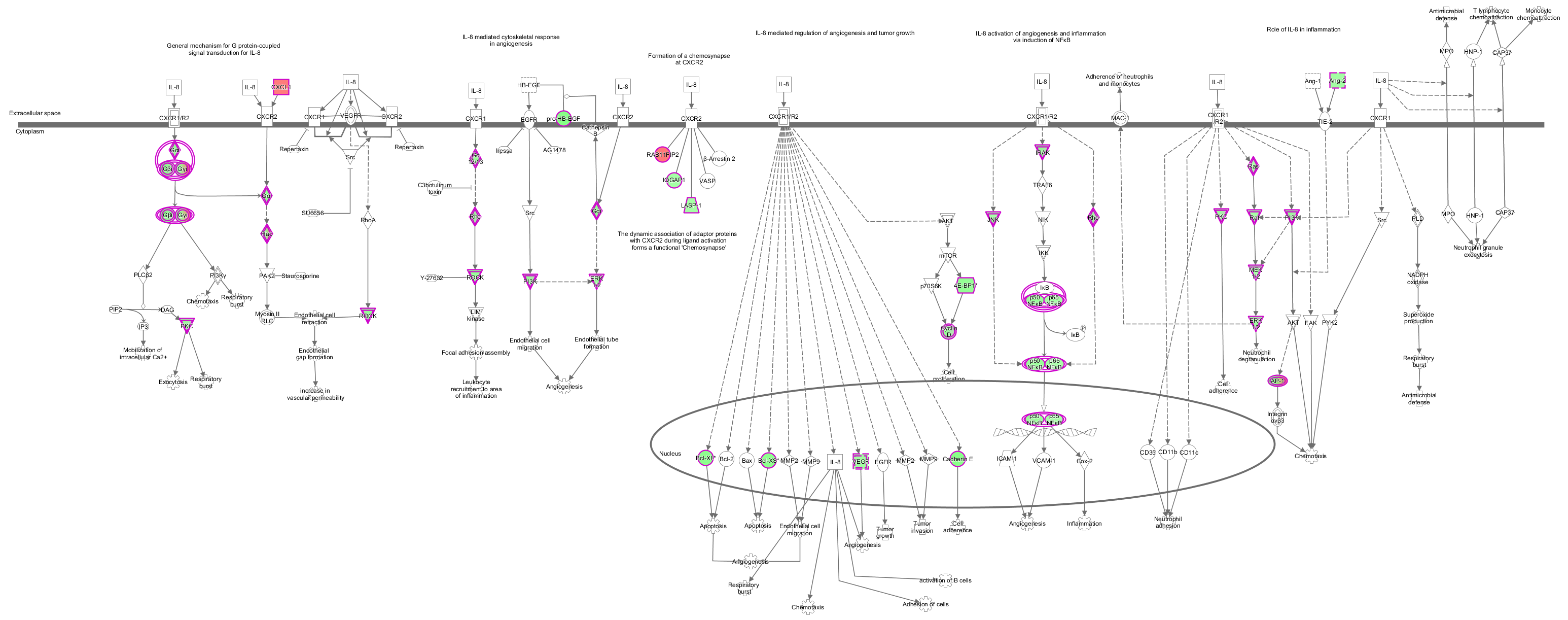

Supplement: Supplementary file 6 — Figure S4. Ingenuity Pathway Analysis depicts IL-8 signaling pathways based on microarray data and available literatures (TIF 515 kb) [file 13046_2018_827_MOESM6_ESM.tif]

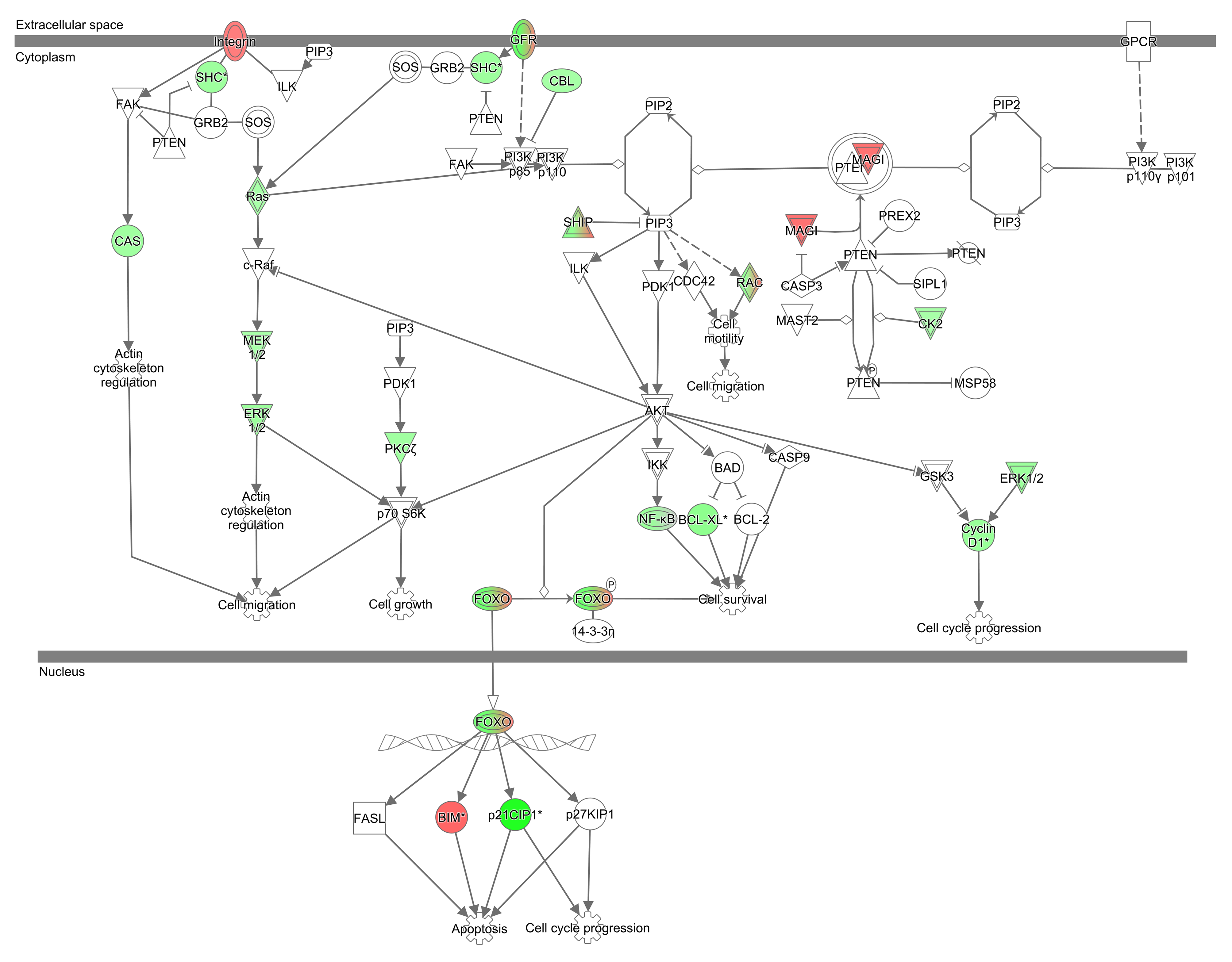

Supplement: Supplementary file 7 — Figure S5. Ingenuity Pathway Analysis depicts PTEN signaling pathways based on microarray data and available literatures (TIF 1204 kb) [file 13046_2018_827_MOESM7_ESM.tif]

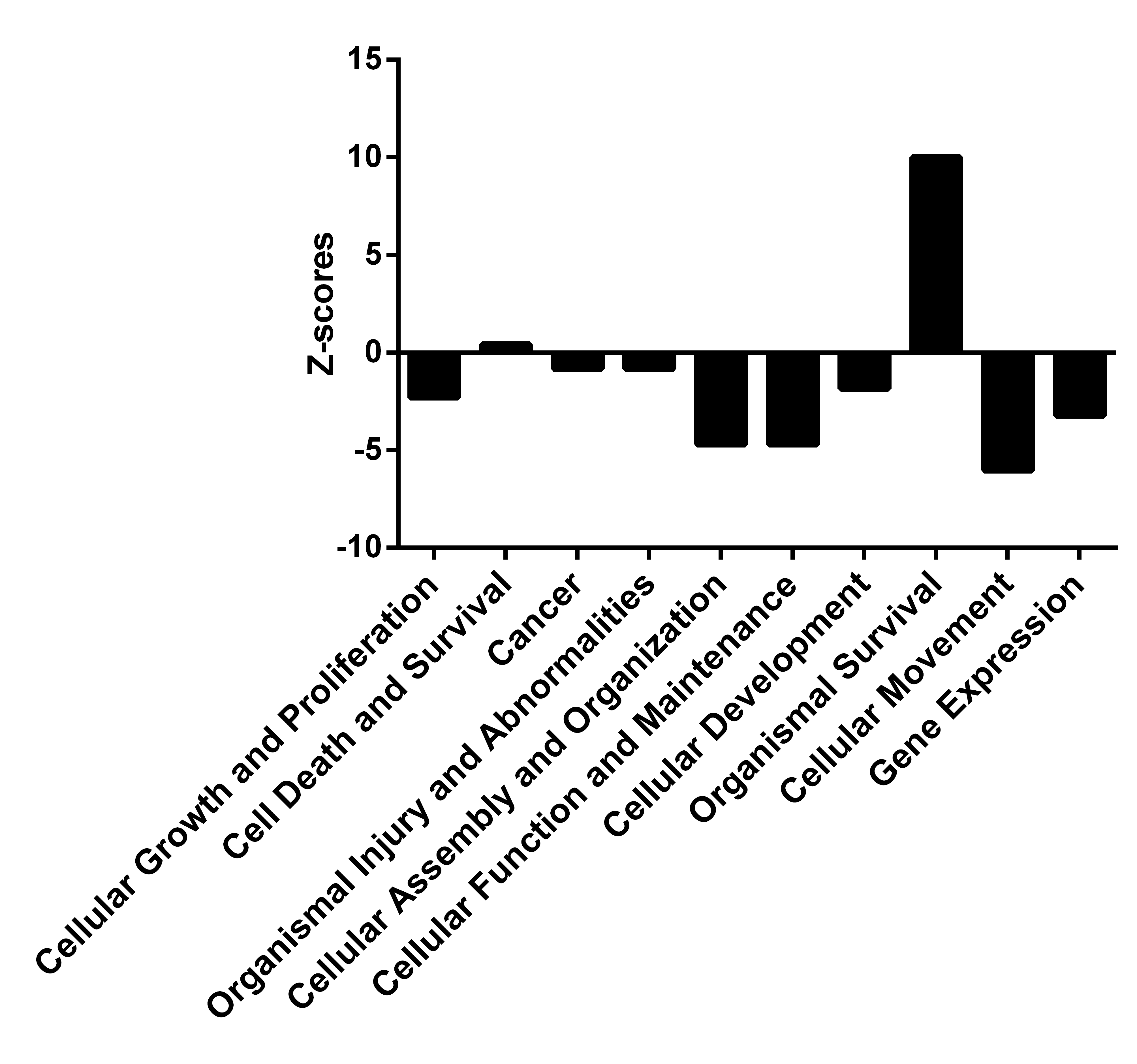

Supplement: Supplementary file 8 — Figure S6. Z-scores of top ten enriched diseases and functions (TIF 305 kb) [file 13046_2018_827_MOESM8_ESM.tif]
