# Supplementary material for: High expression of Ras-related protein 1A promotes an aggressive phenotype in colorectal cancer via PTEN/FOXO3/CCND1 pathway
Source: J Exp Clin Cancer Res. 2018 Jul 31;37:178. doi: 10.1186/s13046-018-0827-y (PMC6069867; doi:10.1186/s13046-018-0827-y)
Supplement: Supplementary file 5 — Table S2. The top ten significantly changed Canonical Pathway based on microarray analysis. (DOC 41 kb) [file 13046_2018_827_MOESM5_ESM.doc]

**Table S2.** The top ten significantly changed Canonical Pathway based on microarray analysis.

| Ingenuity Canonical Pathways | -log(p-value) | Ratio | z-score | Molecules |
| --- | --- | --- | --- | --- |
| Huntington's Disease Signaling | 7.38 | 0.22 | -0.816 | MAP2K4,SGK1,CLTB,HSPA1A/HSPA1B,NAPG,HSPA5,HDAC6,GNB1,CTSD,MAPK3,VAMP3,IRS2, RASA1,GNG12,POLR2L,DNM2,POLR2I,HDAC8,PSMF1,FGFR1,GNG2,CLTC,TBP,HSPA8,BCL2L1, HDAC3,DNAJC5,CLTA,PRKCD,CASP2,CAPN1,GNB2,CAPN11,CASP4,PRKCZ,GNG7,TGM2,SHC1, JUN,SP1,DNAJB1,NAPB,SDHA,GNG4,GNAQ,HSPA2,POLR2G,DNM1,CAPNS1,CYCS,NCOR2, DNM1L,CASP7 |
| Androgen Signaling | 6.97 | 0.279 | -1.941 | RELA,SMAD3,GTF2E2,NFKB1,CCND1,GNG7,PRKCZ,GNB1,SHC1,TGFB1I1,JUN,MAPK3,DNAJB1,POLR2L,GNG12,POLR2I,GNG4,CALR,KAT7,GNAS,GNA12,GNG2,GNAQ,TBP,GTF2F1,POLR2G, CALM1,GNAI3,GTF2E1,PRKCD,GNB2 |
| IL-8 Signaling | 6.44 | 0.223 | -4.867 | MAP2K4,RAC2,RELA,ANGPT2,NFKB1,IQGAP1,CCND1,PRKCZ,GNG7,EIF4EBP1,IRAK1,GNB1,VEGFA,RAB11FIP2,ROCK2,JUN,RHOG,RHOB,CCND3,MAP2K2,MAPK3,CXCL1,IRS2,RHOF,GNG12, LASP1,GNG4,GNAS,GNA12,FGFR1,GNG2,RAC1,HBEGF,BCL2L1,GNAI3,FOS,CDH1,RRAS2,RHOQ,ARAF,PRKCD,GNB2,IRAK4,FNBP1 |
| Ephrin B Signaling | 6.35 | 0.315 | -2.111 | GNG4,RAC2,EPHB4,GNAS,CXCR4,GNA12,GNG2,GNAQ,RAC1,GNG7,ROCK2,GNB1,EPHB6,  GNAI3,EFNB2,CBL,CFL2,ABI1,EFNB1,MAPK3,GNB2,ITSN2,GNG12 |
| Unfolded protein response | 6.18 | 0.352 | NaN | PPARG,CALR,P4HB,INSIG1,HSPA1A/HSPA1B,CEBPD,CEBPB,HSPA5,HSPA2,CEBPG,MBTPS2, SEL1L,HSPA8,HSP90B1,UBXN4,SREBF1,ERO1B,VCP,CEBPA |
| Signaling by Rho Family GTPases | 5.85 | 0.202 | -3.893 | MAP2K4,SEPT9,SLC9A1,CLIP1,ROCK2,GNB1,RHOG,MAP2K2,CFL2,RHOB,EZR,MAPK3,IRS2,CDH13,GNG12,FGFR1,GNG2,RAC1,CDH1,RHOQ,GNB2,FNBP1,RELA,ARPC1B,SEPT11,NFKB1,IQGAP1,PRKCZ,GNG7,JUN,PPP1R12A,ARHGEF2,ARHGEF3,RHOF,PI4KA,MYL12A,GNG4,SEPT5,NEDD4,GNAS,GNA12,ITGA2,GNAQ,GNAI3,FOS,WIPF1,CDH17,SEPT6,SEPT2,MSN |
| PTEN Signaling | 5.73 | 0.252 | 1.671 | RAC2,FOXO4,RELA,BMPR2,NFKB1,BCAR1,CCND1,OCRL,PRKCZ,TGFBR2,SHC1,MAP2K2,MAPK3,FOXO3,CSNK2B,FGFR1,ITGA2,RAC1,INPP5D,DDR1,SYNJ2,BCL2L1,CBL,RRAS2,FOXO1, CDKN1A,INPP5K,FGFRL1,BCL2L11,MAGI3 |
| Sertoli Cell-Sertoli Cell Junction Signaling | 5.57 | 0.219 | NaN | MAP2K4,SPTBN1,TUBA1B,CLDN7,TUBB,BCAR1,JUN,MAP2K2,CLDN4,MAPK3,CGN,MAP3K7,TUBB4A,TUBA1C,MTMR2,JUP,VCL,ACTN1,EPN1,TJP2,TJP1,ITGA2,RAC1,TUBA4A,EPB41,F11R,CDH1,TJP3,MAPK14,RRAS2,CLDN12,CLDN1,MAP3K20,SPTAN1,ACTN4,CLINT1,MAP3K3,CLDN3, NECTIN2 |
| Phospholipase C Signaling | 5.29 | 0.198 | -3.772 | RELA,MPRIP,RALA,RPS6KA3,PPP1CB,NFKB1,PRKCZ,GNG7,HDAC6,TGM2,GNB1,SHC1,LCK,NFAT5,RHOG,RHOB,PPP1R12A,MAP2K2,MAPK3,ARHGEF2,ARHGEF3,RHOF,PPP3CA,GNG12,MYL12A,GNG4,GNAS,HDAC8,ITGA2,RALB,GNG2,CHP1,RAC1,GNAQ,NFATC4,RAP1A,CALM1, HDAC3, PLA2G4D, RRAS2, RHOQ, SYK, PRKCD, LAT, GNB2, LYN,FNBP1 |
| HIPPO signaling | 5 | 0.267 | 0.535 | TJP2,YWHAB,RASSF6,SMAD3,WWTR1,PPP1CB,PPP1R11,LATS2,DLG3,PRKCZ,PPP1CC,PPP1R12A,WWC1,AJUBA,BTRC,PPP2R2C,PPP2R5C,PPP2R5E,SFN,SMAD1,AMOT,LATS1,RASSF1 |
